# Structure and Luminescence Properties of Transparent Germanate Glass-Ceramics Co-Doped with Ni^2+^/Er^3+^ for Near-Infrared Optical Fiber Application

**DOI:** 10.3390/nano11082115

**Published:** 2021-08-19

**Authors:** Magdalena Lesniak, Marcin Kochanowicz, Agata Baranowska, Piotr Golonko, Marta Kuwik, Jacek Zmojda, Piotr Miluski, Jan Dorosz, Wojciech Andrzej Pisarski, Joanna Pisarska, Dominik Dorosz

**Affiliations:** 1 Faculty of Materials Science and Ceramics, AGH University of Science and Technology, 30 Mickiewicza Av., 30-059 Krakow, Poland; ddorosz@agh.edu.pl; 2 Faculty of Electrical Engineering, Bialystok University of Technology, 45D Wiejska Street, 15-351 Bialystok, Poland; m.kochanowicz@pb.edu.pl (M.K.); p.golonko@doktoranci.pb.edu.pl (P.G.); j.zmojda@pb.edu.pl (J.Z.); p.miluski@pb.edu.pl (P.M.); doroszjan@pb.edu.pl (J.D.); 3 Faculty of Mechanical Engineering, Bialystok University of Technology, 45C Wiejska Street, 15-351 Bialystok, Poland; a.baranowska@pb.edu.pl; 4 Institute of Chemistry, University of Silesia, 9 Szkolna Street, 40-007 Katowice, Poland; marta.kuwik88@gmail.com (M.K.); wojciech.pisarski@us.edu.pl (W.A.P.); joanna.pisarska@us.edu.pl (J.P.)

**Keywords:** glass-ceramics, Ni^2+^/Er^3+^, luminescence, structure, microstructure, ZnGa_2_O_4_

## Abstract

An investigation of the structural and luminescent properties of the transparent germanate glass-ceramics co-doped with Ni^2+^/Er^3+^ for near-infrared optical fiber applications was presented. Modification of germanate glasses with 10–20 ZnO (mol.%) was focused to propose the additional heat treatment process controlled at 650 °C to obtain transparent glass-ceramics. The formation of 11 nm ZnGa_2_O_4_ nanocrystals was confirmed by the X-ray diffraction (XRD) method. It followed the glass network changes analyzed in detail (MIR—Mid Infrared spectroscopy) with an increasing heating time of precursor glass. The broadband 1000–1650 nm luminescence (λ_exc_ = 808 nm) was obtained as a result of Ni^2+^: ^3^T_2_(^3^F) → ^3^A_2_(^3^F) octahedral Ni^2+^ ions and Er^3+^: ^4^I_13/2_ → ^4^I_15/2_ radiative transitions and energy transfer from Ni^2+^ to Er^3+^ with the efficiency of 19%. Elaborated glass–nanocrystalline material is a very promising candidate for use as a core of broadband luminescence optical fibers.

## 1. Introduction

The rapid increase in the capacity of modern information technology (IT) based on the computer network and optical communications demands optical fiber amplifiers with the ultrabroad and efficient gain bandwidth in the telecommunication windows. For this reason, the possible expansion of emission bands in erbium-doped fiber amplifiers (EDFA) in different ways is a hot topic [1]. In particular, the following co-doping system of rare-earth ions (RE) has been proposed, including: Er^3+^/Tm^3+^, Pr^3+^/Er^3+^, Er^3+^/Tm^3+^/Pr^3+^, Yb^3+^/Er^3+^/Tm^3+^ and others [2,3,4,5]. However, the main general problem in co-doping systems is the narrowing of 4f-4f transitions and unwanted energy transfer mechanism between RE ions which strongly limits the effective emission in the near-infrared spectral range. It is well-known that possible energy transfer channels depend on the phonon energy of the host and the type of electrostatic interionic interactions [6,7,8]. In the case of low-phonon glasses, the upconversion mechanisms (ETU—energy transfer upconversion, ESA—excited state absorption) leads to the depopulation of higher energy levels, and then excitation energy is partially distributed between rare-earth ions [9]. From the other side, in matrices with high-phonon energy, where the upconversion processes are negligible, the cross-relaxation and energy migration mechanisms are more prominent, and finally, the luminescence quenching is observed [10,11] In order to minimize the above limitations, great efforts have been put into the combination of the transition metal (TM) and RE ions in glasses and glass-ceramics. Transitions metals, such as Ni^2+^ ions, show the broadband emission in the near-infrared spectral range when they are localized in a crystal field environment [12]. However, from a practical point of view, it is impossible to fabricate optical fibers from ceramics. Thus, to realize efficient ultrabroad emission in the NIR region, the development of transparent glass-ceramics with embedded nanocrystals doped with transition metals and rare-earth ions is now the challenge in optical fiber technology. 

One of the most promising multiple active ions for potential broad NIR luminescence are Ni^2+^/Er^3+^ ions. Recently, Zhou et al. showed that broadband NIR luminescence in the wavelength region of 1200–1600 nm is possible to obtain in silicate glass-ceramics by the manipulation of the energy transfer process between dopants selectively incorporated into the specified nanocrystals of γ-Ga_2_O_3_ and hexagonal LaF_3_ [13]. A similar effect was observed by Zhang in Ni^2+^/Er^+^ dual-doped transparent glass-ceramics containing γ-Ga_2_O_3_ and β-YF_3_ nanocrystals [14]. As a result of the subsequent heat-treatment process, the broadband NIR emission (originating from Ni^2+^: ^3^T_2_(F) → ^3^A_2_(F) and Er^3+^: ^4^I_13/2_ → ^4^I_15/2_) was realized in SiO_2_–Al_2_O_3_–Ga_2_O_3_–YF_3_–LiF glass-ceramics under 980 nm laser excitation. Zheng et al. suggest that the key factor for efficient NIR luminescence is the selective partition of the two species of optically active ions into different nanocrystal phases. In his work, the Ni^2+^/Er^3+^ co-doped transparent gallium-silicate glass-ceramics containing ZnGa_2_O_4_ nanocrystals show a super-broadband luminescence with the full width at half maximum (FWHM) about 400 nm [15]. Another pointed out the important issue is the selection of the optimal host both with high RE ions solubility and fiberization features. Silicate-based glass-ceramics have relatively high phonon energy (1100 cm^−1^) and high melting temperature (1600 °C) which significantly decreases the quality of the initial glasses [16,17]. Compared with silica glass, germanate glasses are characterized by excellent glass-forming ability, lower phonon energy (~850 cm^−1^), higher RE ions solubility, and refractive index above 1.75 [18,19,20]. It should be noted that the higher refractive index of the germanate-based glasses leads to lower scattering losses, resulting in a decreasing difference in refractive index between nanocrystals and glass matrix. Besides, partial substitution of SiO_2_ by GeO_2_ in gallium-silicate glasses leads to a lower melting temperature and minimizes the non-radiative transitions [21]. The combination of glass-forming elements of different phonon energy also results in greater separation of optically active centers and enables effective broadening of the spontaneous emission spectrum [22]. 

Regarding the above considerations, in our study, we developed GeO_2_-Ga_2_O_3_-ZnO-K_2_O glass and then glass-ceramics co-doped with Ni^2+^/Er^3+^ ions as a potential material for optical fiber with broadband NIR luminescence. Optimal glass composition was selected by subsequent substitution of GeO_2_ by ZnO. The controllable heat-treatment process with rigorous time conditions was designed to obtain ZnGa_2_O_4_ nanocrystals in the vicinity of Ni^2+^ ions. The structural properties of precursor glass and a glassy matrix of fabricating gallo-germanate glass-ceramics were analyzed by Fourier-transform infrared (FTIR) measurements in detail. In particular, luminescence spectra and decay curves were examined for glass and glass-ceramic samples as a function of different concentrations of ZnO and the annealing time. Analysis of the energy transfer mechanism between Ni^2+^ and Er^3+^ ions showed that the investigated glass-ceramics are promising candidates for their application in broadband emission sources of radiation or optical amplifiers.

## 2. Materials and Methods

**Figure 1:**
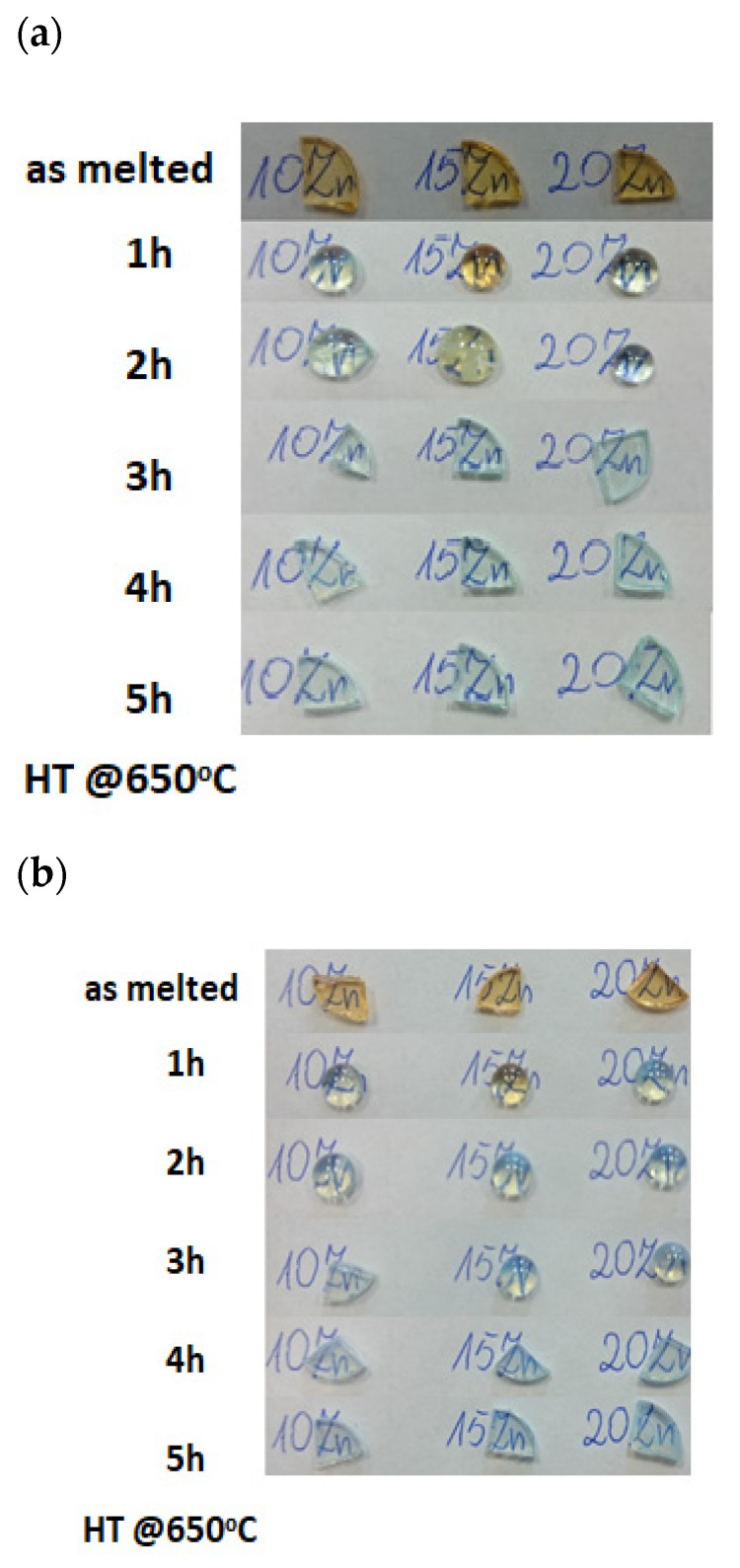
Image of the GGK with 10, 15, 20 ZnO glass and glass-ceramics (HT@650 °C for 1–5 h) (**a**) doped with 0.1 NiO, (**b**) co-doped with 0.1 NiO/0.1 Er_2_O_3_.

The germanium based GGK glasses were prepared according to the following general molar formula: (1-x-y)75GeO_2_-10Ga_2_O_3_-xZnO-5K_2_O-yNiO-0.1Er_2_O_3_, (x = 10, 15, 20, y = 0, 0.1%) by standard melting and quenching method. The homogenized set (purity of materials 99.99% Sigma-Aldrich, Saint Louis, MO, USA) was melted in a platinum crucible in an electric furnace (CZYLOK Company, Jastrzębie-Zdrój, Poland) at T = 1500 °C for 30 min. The molten glass was poured out onto a stainless-steel plate and then annealed in an air atmosphere at 560 °C for 12 h. Glass-ceramic materials were obtained by additional heat treatment of the as-melted glasses in Czylok tube furnace at 650 °C for 1–5 h. The glasses and glass-ceramics samples were labeled according to the ZnO amount (10ZnO, 15ZnO) and heating time (3 h and 5 h at 650 °C). The photos of obtained samples were presented in Figure 1a,b.

FTIR spectra were recorded with a Bruker Company Vertex 70v spectrometer (Rheinstetten, Germany). The MIR spectra were normalized to the highest peak. FTIR spectra have been decomposed using Fityk software (0.9.8 software, open-source (GPL2+)) based on the analysis of second derivatives with different degrees of smoothing. The coefficient of determination was 0.99. The standard deviation of the position of each of the component bands was ±4 cm^−1^.

The excitation wavelength of 532 nm was used, and power was about 10 mW. Acquisition time was set to 30 seconds. The formed crystallites were examined by the X-ray powder diffraction (XRD) method in the range of 10° to 90° using an X’Pert Pro diffractometer (PANalytical, Eindhoven, The Netherlands). The Cu X-ray tube with K_α_ radiation was used. DSC measurement was performed with 10 K/min using the SETARAM Labsys thermal analyzer (Setaram Instrumentation, Caluire, France). The glass-ceramics morphology was observed by an FEI Company Nova Nano SEM 200 scanning electron microscope (Hillsboro, OR, USA) and the analyses were performed in the secondary electron mode (SE). The absorbance and emission spectra of glasses in the range of 1 μm were measured using the Stellarnet Green-Wave spectrometer (Setaram Instrumentation, Caluire, France) and a high-power LIMO laser diode (*λ_p_* = 808 nm, P_opt (max)_ = 0.1–30 W). Spectral measurements in the range of 1000–1700 nm were carried out using Acton 2300 i monochromator (Acton Research Corporation, Acton, MA, USA) equipped with an InGaAs detector. 

## 3. Results and Discussion

### 3.1. Structural Properties

**Figure 2:**
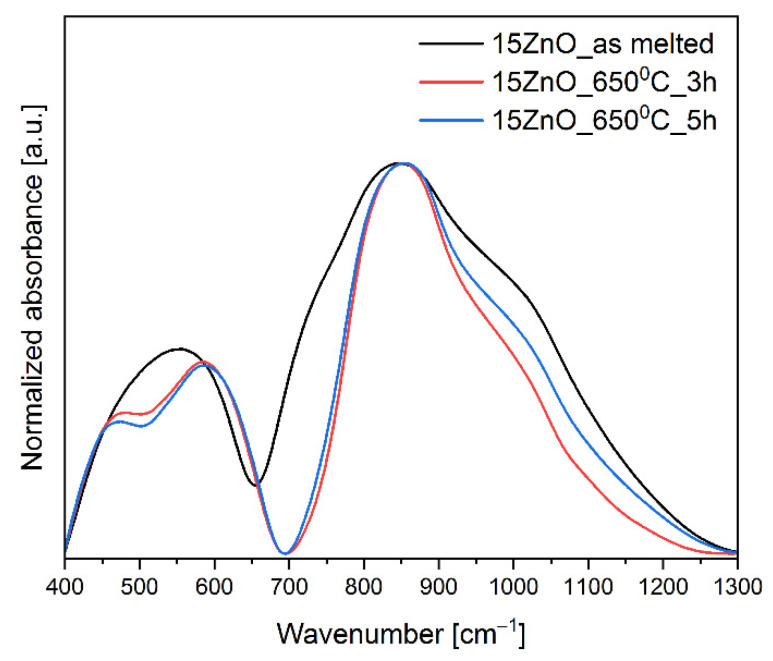
MIR spectra of the glass and glass-ceramics co-doped with Ni^2+^ and Er^3+^.

In Figure 2, MIR spectra of 15 ZnO glass, and glass-ceramics samples doped with Ni^2+^ and Er^3+^ in the 400–1300 cm^−1^ range were presented. Due to the low-resolution technique, the presence of the nanocrystals has not been detected in spectra of glass-ceramics samples (lack of the bands in MIR spectra assigned to the bonds in crystals lattice). 

**Figure 3:**
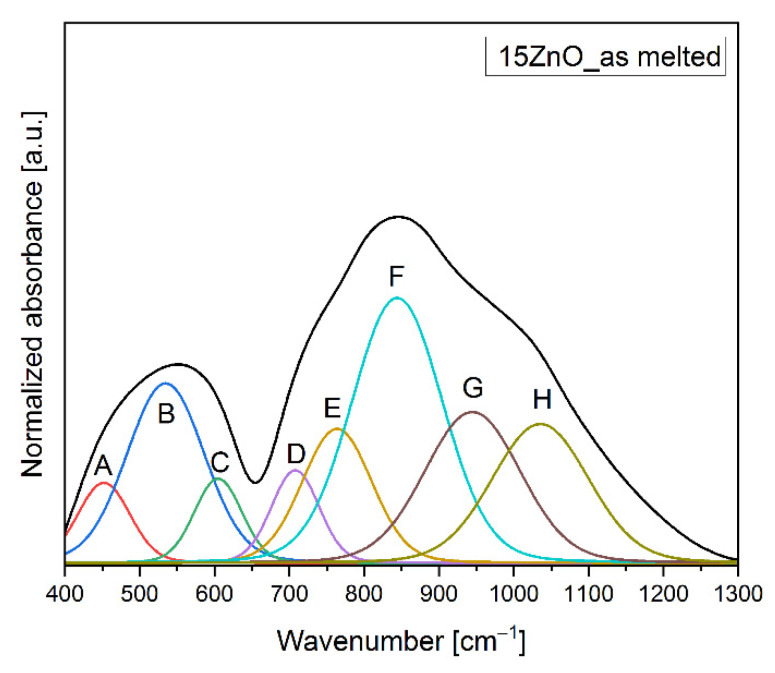
MIR spectrum of the 15ZnO glass co-doped with Ni^2+^ and Er^3+^.

**Figure 4:**
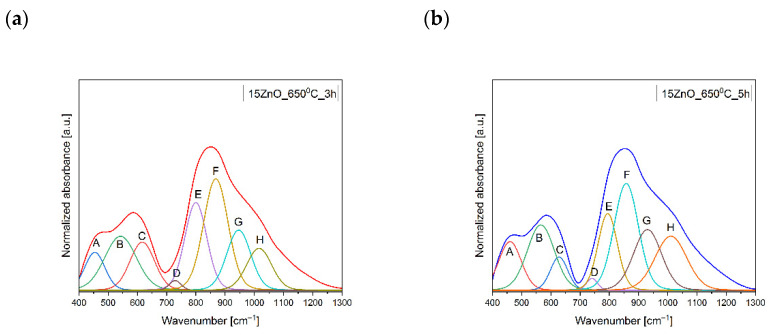
MIR spectrum of the 15ZnO glass-ceramics sample obtained after HT@650C for (**a**) 3 h, (**b**) 5 h.

In the MIR spectra, the two broad bands in the ranges of 400 cm^−1^–700 cm^−1^, and 700 cm^−1^–1300 cm^−1^ were observed. In order to comprehend the changes in the matrix network of the glass and glass-ceramics co-doped with Ni^2+^ and Er^3+^, the MIR spectra have been decomposed on component bands and presented in Figure 3 and Figure 4a,b. The parameters and assignment of the components bands in the MIR spectra have been shown in Table 1 and Table 2, respectively. 

The decomposed MIR spectra of the glass and glass-ceramics co-doped with Ni^2+^ and Er^3+^ showed eight bands, such as at ~450–460 cm^−1^ (A), 530–560 cm^−1^ (B), 600–630 cm^−1^ (C), 700–740 cm^−1^ (D), 760–800 cm^−1^ (E), 840–870 cm^−1^ (F), 930–950 cm^−1^ (G), and 1010–1040 cm^−1^ (H) (Figure 3 and Figure 4a,b). The band at 450–460 cm^−1^ (A) was due to symmetrical stretching bonds of Ge^[4]^, Ga^[4]^-O,-Ge^[4]^, Ga^[4]^ in germano(gallo)oxygen four-membered rings. The band at 530–560 cm^−1^ (B) might be associated with the symmetrical stretching bonds of Ge^[4]^, Ga^[4]^-O-Ge^[4]^, Ga^[4]^ in the three-membered germanano(gallo)oxygen rings. The band at 600–630 cm^−1^ (C) was attributed to the symmetrical stretching bonds of Ge^[4]^, Ga^[4]^-O-Ge^[4]^, Ga^[4]^. The band at 700–740 cm^−1^ (D) was assigned to symmetrical stretching bonds of Ge^[6]^, Ga^[6]^-O-Ge^[6]^, Ga^[6]^. The asymmetrical stretching bonds of Ge^[4]^,Ga^[4]^-O-Ge^[4]^,Ga^[4]^ connecting [GeO]_4_/[GaO]_4_ tetrahedrons in 760–800 cm^−1^ (E) were detected. The band at ~840–870 cm^−1^ (F) showed up due to the asymmetrical stretching vibrations of the Ge-O-Ge, and Ga-O-Ga connecting [GeO_4_], [GaO_4_] tetrahedra, respectively. The band at around 930–950 cm^−1^ (G) was assigned to the Ge–O–Ge or Ga–O–Ga stretching vibrations in [GeO_4_]/[GaO_4_]. Additionally, the band at 1010–1040 cm^−1^ might be associated with the stretching vibrations of the (Ge-O^−^) of GeO_4_ units [23,24,25,26,27,28,29,30,31,32,33,34].

The decomposed MIR spectra showed that heating of the GGK with 15ZnO glass during 3h caused a growth in the polymerization of the glass network. It might be seen in Figure 2 and Figure 3, and Table 1 that the integral intensity of the A, B, C, E, and F bands raised after the following thermal treatments: from 14–15ZnO glass to 25–15ZnO_650 °C_3 h, from 45–15ZnO glass to 55–15ZnO_650 °C_3 h, from 13–15ZnO glass to 38–15ZnO_650 °C_3 h, from 30–15ZnO glass to 62–15ZnO_650 °C_3 h and 78–15ZnO glass 87–15ZnO_650 °C_3 h, respectively. Moreover, when the glass was heated at 650 °C in 3 h, a reduction in the intensity of band due to symmetrical stretching bonds of Ge^[6]^, Ga^[6]^-O-Ge^[6]^, Ga^[6]^ (from 14–15ZnO glass to 4–15ZnO_650 °C_3 h) was observed (Figure 3, and Table 1 and Table 2). A different situation was observed for the 15ZnO_650 °C_5h sample. With the increase in the heating time of glass doped with nickel and erbium, the reduction of the order degree system was noticed. This was confirmed by the integral intensity reduction of the following B, C, E, F, G and H bands from 45–15ZnO glass to 30–15ZnO_650 °C_5 h, from 13–15ZnO glass to 10–15ZnO_650 °C_5 h, from 30–15ZnO glass to 25–15ZnO_650 °C_5 h, from 78–15ZnO glass to 44–15ZnO_650 °C_5 h, from 47–15ZnO glass to 29–15ZnO_650 °C_5 h and 44–15ZnO glass 29–15ZnO_650 °C_5 h, respectively. It might be related to the incorporation of the part of the Ni^2+^ and Er^3+^ ions into the crystalline phase [24].

**Figure 5:**
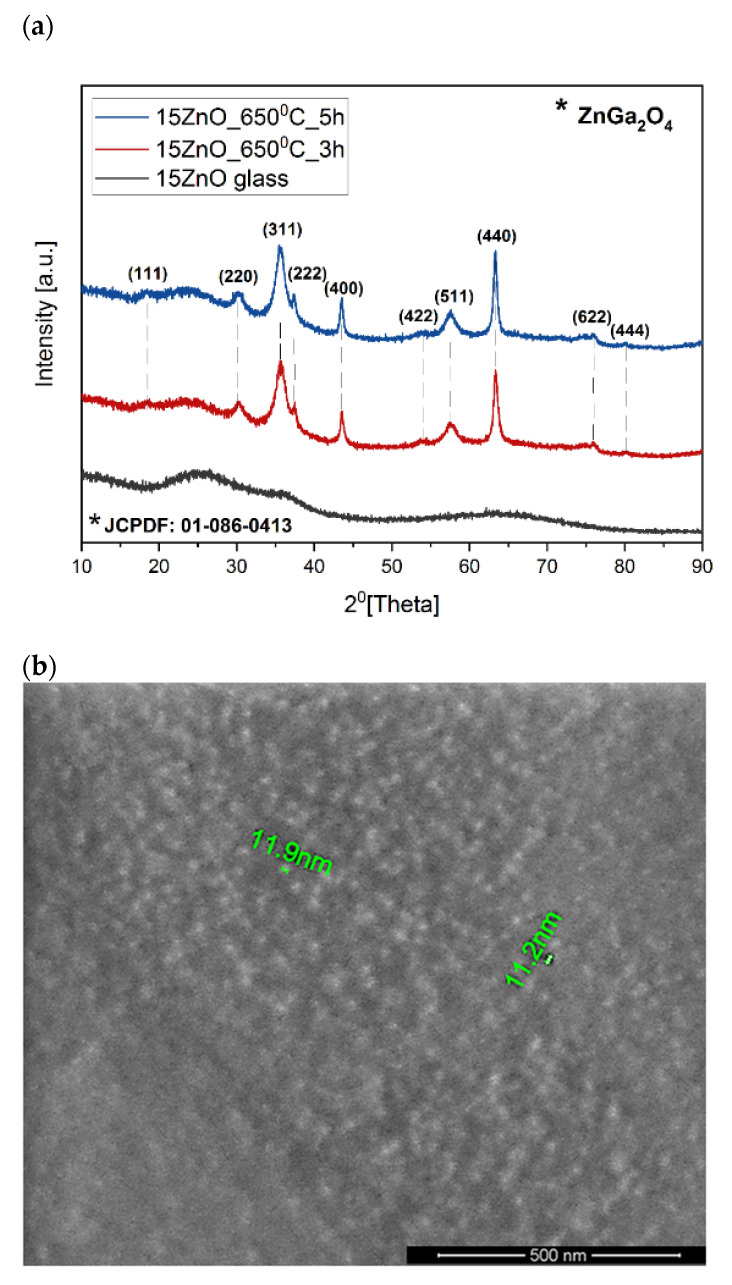
(**a**) Diffraction patterns of the samples doped with nickel and erbium ions doped with 0.1NiO, (**b**) SEM image of the GGK with 15ZnO co-doped with 0.1NiO/0.1Er_2_O_3_ glass-ceramics (HT@650 °C for 1–5 h) with indicated ZnGa_2_O_4_ nanocrystals.

As the nanocrystals have not been observed by the MIR spectrum the diffraction patterns of the samples were measured (Figure 5a). The amorphous state of the 15ZnO glass sample has been confirmed by a broad diffraction hump in the range of 2θ from 15° to 40°. In the diffraction patterns of the glass heated at 650 °C in three (15ZnO_6500C_3h) and five (15ZnO_6500C_5 h) hours, the peaks were observed. They were assigned to the zinc gallium oxide (ZnGa_2_O_4_) cubic phase (JCPDF: 01-086-0413) [35]. The average size of the nanocrystals was ca. 11 nm (seen also at SEM Figure 5b) which is much smaller than the visible light wavelength. Thus, obtained glass-ceramics are transparent (Figure 1a,b).

The size of the ZnGa_2_O_4_ nanocrystals presented in Figure 5b was also checked according to the calculation by Scherrer’s Equation (1) [36,37]. The obtained results for 15ZnO_650 °C_3h and 15ZnO_650 °C_5h samples have been presented in Table 3 and Table 4. The crystalline size of crystals has been evaluated for diffraction peaks visible in Figure 5a. According to the calculated data, the crystalline size of nanocrystals is in the 3–18 nm range for both samples. These data are in line with the SEM results and give an overview of the crystal size distribution in the glass-ceramic samples. Moreover, the longer heating time of Ni/Er co-doped glass sample from 3 h to 5 h did not cause an increase in crystal size. The difference in the size of the crystallites for the analyzed samples is within the measurement bar (±2 nm). This suggests that with an increase in heating time of the glass sample, the number of nanocrystallines grew [38].

(1) $$d=\frac{k\lambda }{\beta \cos\theta }$$

where:

*k*—shape factor (0.9) [-];
*λ*—wavelength of X-ray [0.154056 nm];
*Β*—diffraction peak broadening at half the maximum intensity (FWHM) [rad];
*θ*—Bragg’s angle [°].

**Figure 6:**
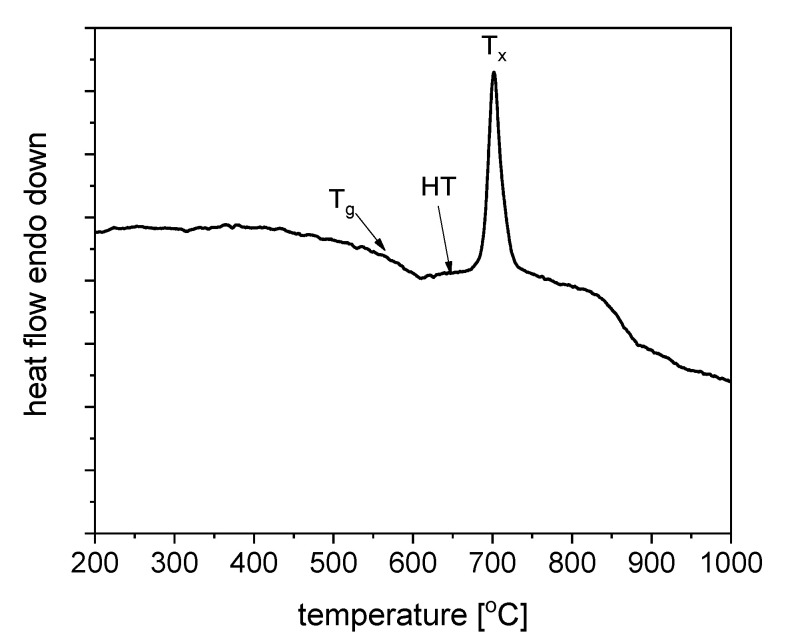
DSC curve of fabricated GGK with 15ZnO glass with heating rate 10 °C/min.

Thermal treatment conditions have been investigated using the DSC analysis of the as-melted precursor GGK glass at a heating rate of 10 °C/min and shown in Figure 6. One exothermic, crystallization peak located around 703 °C was observed. The glass transition temperature T_g_ and the onset of the crystallization temperature were about 560 °C and 675 °C, respectively. The thermal stability parameter ΔT defined as T_x-_T_g_ was calculated to be 115 °C. It proofs the good thermal stability of glass, which is essential in the case of controllable crystallization of glasses and fabrication of the glass-ceramics material with embedded nanocrystals. Moreover, optical fiber technology ΔT above 100 °C describes glass as a suitable material for the optical fiber drawing [39].

### 3.2. Optical Properties

**Figure 7:**
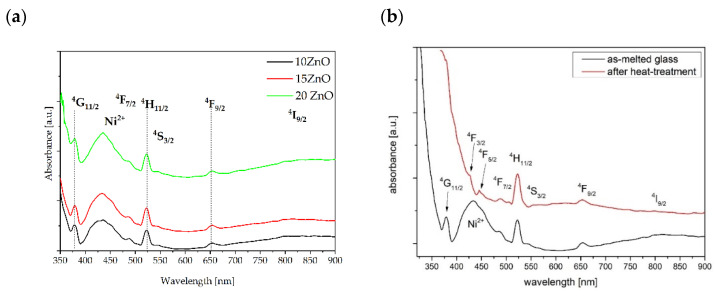
Absorbance spectra of fabricated GGK glasses co-doped with 0.1NiO/0.1Er_2_O_3_ and: (**a**) different molar concentration of ZnO, (**b**) 15ZnO before (black line) and after (red line) controllable heat treatment (HT) process at 650 °C for 5 h.

The absorbance spectra of 0.1NiO/Er_2_O_3_ co-doped glasses with 10, 15, and 20 mol% ZnO is shown in Figure 7a. The spectra consist of five bands that are assigned to the transition from the ground state of Er^3+^ to the ^4^G_11/2_, ^4^F_7/2_, ^4^H_11/2_, ^4^F_9/2_, ^4^I_9/2_ higher levels, and one band at 430 nm which is derived from the transition of ^3^A_2_(^3^F) → ^3^T_1_ (^3^P) in five folded Ni^2+^ ions. It should be noted that the presence of ZnO does not affect the background absorption. However, the shape of the absorption spectrum has changed after the heat treatment process (Figure 7b). It was also proofed by changing the color of the glass and glass-ceramics (Figure 1a,b). This effect is related to the variation of the Ni^2+^ ions in sites: from tetrahedral in glasses to octahedral in glass-ceramics [40,41]. Therefore, the Ni^2+^ ions should be embedded in the ZnGa_2_O_4_ nanocrystals after the heat treatment. It was reported in the literature that blue, brown, and yellow-green glass colors are observed in the case of ^4^Ni^2+^, ^5^Ni^2+^, and ^6^Ni^2+^ coordination, respectively [42].

Besides, after heat treatment at 650 °C for 5 h, UV absorption is red shifted and background absorption is slightly higher in comparison to as-melted glass. A similar effect was reported in the literature [15].

**Figure 8:**
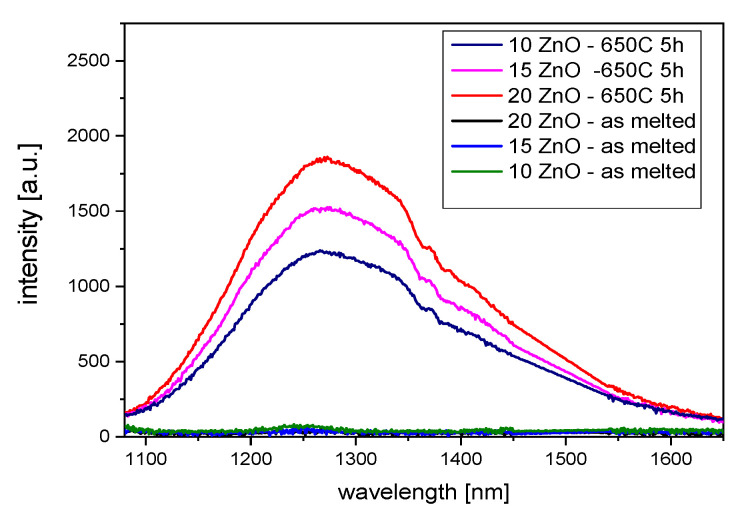
Infrared luminescence spectra of 0.1NiO doped GGK with glass and transparent glass-ceramics with 10, 15, and 20 ZnO (mol.%), (HT@650 °C for 5 h), λ_exc_ = 808 nm.

In Figure 8, luminescence spectra of the GGK glass and glass-ceramics with 10, 15, and 20 ZnO (mol.%) doped with 0.1NiO (λ_exc_ = 808 nm) are presented. The broadband 1050–1650 nm emission centered at 1280 nm can be assigned to the ^3^T_2_(^3^F) → ^3^A_2_(^3^F) transition of octahedral Ni^2+^ ions in the ZnGa_2_O_4_ nanocrystals [43]. It is seen that the emission spectrum is composed of two broad sub-peaks located at 1280 nm and 1460 nm. It is known that ZnGa_2_O_4_ is a two-site spinel compound [33]. It was also found that luminescence intensity increases with the ZnO content. It might be the result of the increase of Ni^2+^ in octahedral sites with the ZnGa_2_O_4_ nanocrystals [12]. These results correspond well to the further discussed emission decay curves.

**Figure 9:**
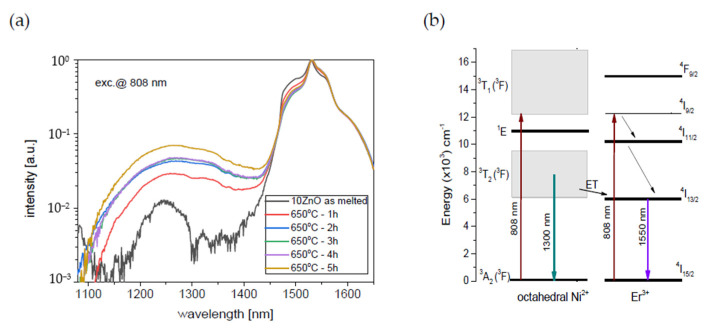
(**a**) Infrared luminescence spectra of 0.1NiO/0.1Er_2_O_3_ co-doped GGK glass and transparent glass-ceramics with 10 ZnO (mol.%), (HT@650°C for 1–5 h), (**b**) simplified energy level diagram of the Ni^2+^ and Er^3+^ ions in the GGK glass-ceramics host with possible transitions and Ni^2+^ to Er^3+^ energy transfer, λ_exc_ = 808 nm.

**Figure 10:**
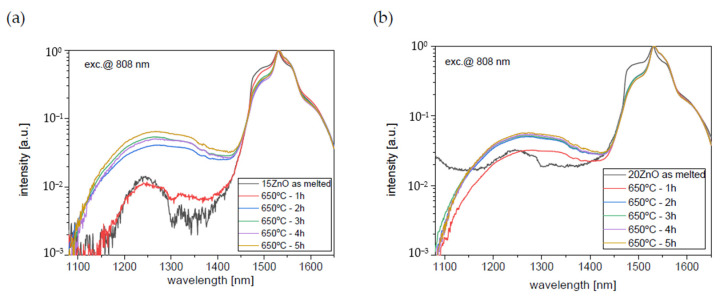
Infrared luminescence spectra of 0.1NiO/0.1Er_2_O_3_ co-doped GGK glass and transparent glass-ceramics with (**a**) 15 ZnO (mol.%), (**b**) 20 ZnO (mol.%), (HT@650 °C for 1–5 h), λ_exc_ = 808 nm.

**Figure 11:**
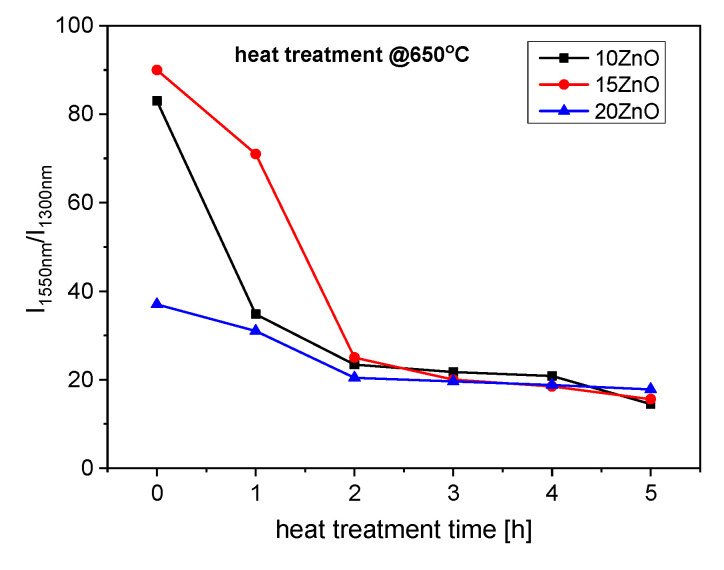
I_1550nm_/I_1300nm_ intensity ratio of 0.1NiO/0.1Er_2_O_3_ co-doped GGK glass and transparent glass-ceramics with 15 ZnO (mol.%), 20 ZnO (mol.%), (HT@650 °C for 1–5 h), λ_exc_ = 808 nm.

**Figure 12:**
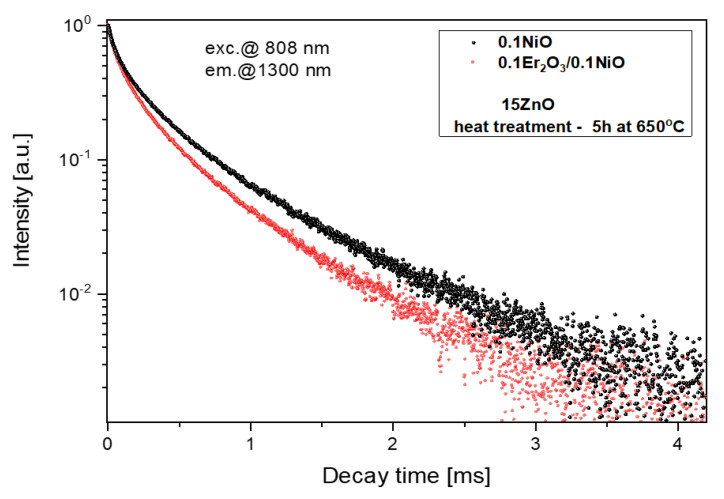
Luminescence decay curves from Ni^2+^:^3^T_2_(^3^F) level, λ_exc_ = 808 nm.

**Figure 13:**
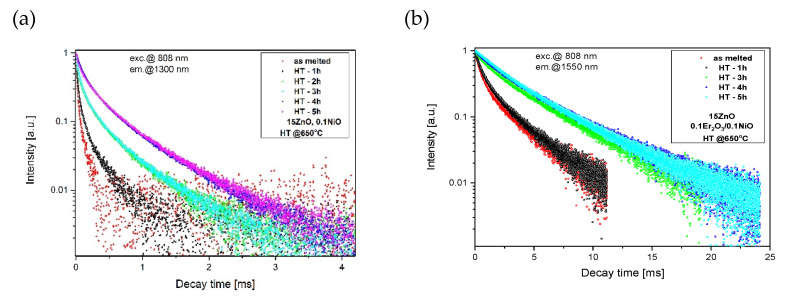
Luminescence decay curves from (**a**) Ni^2+^:^3^T_2_(^3^F), (**b**) Er^3+^:^4^I_13/2_ levels in GGK with 15 ZnO glass and glass-ceramics (HT@650 °C for 1–5 h), λ_exc_ = 808 nm.

**Figure 14:**
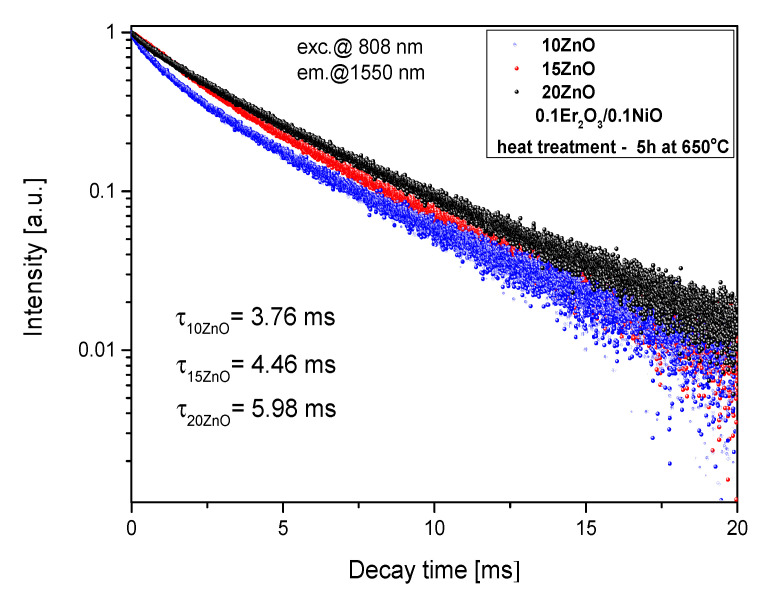
Luminescence decay curves from Er^3+^:^4^I_13/2_ level in 5, 10, 15 ZnO glass-ceramics (HT@650 °C for 5 h), λ_exc_ = 808 nm.

To investigate ultra-broadband emission in the range of 1050–1650 nm GGK glass was co-doped with 0.1NiO/0.1Er_2_O_3_. The effect of the heat treatment of GGK glasses with 10, 15, 20 ZnO on the luminescence spectra under 808 nm laser excitation is presented in Figure 9 and Figure 10. Moreover, the influence of the heat treatment time on the I_1550nm_/I_1300nm_ intensity ratio has been presented in Figure 11. Emission spectra in all investigated glass-ceramic samples are the result of a superposition of the 1280 nm and 1550 nm luminescence bands. Ni^2+^: ^3^T_2_(^3^F) level is populated directly by the ground-state absorption of the 808 nm laser pump whereas erbium ions also directly absorb the same excitation wavelength. Next, within the Er^3+^: ^4^I_9/2_ → ^4^I_11_/_2_ → ^4^I_13/2_ the nonradiative relaxation occurs. At the same time, part of the energy is transferred from Ni^2+^: ^3^T_2_(^3^F) to Er^3+^: ^4^I_13/2_ level. It was confirmed by analysis of the luminescence decay curves (Figure 12, Figure 13 and Figure 14). Finally, the 1280 nm luminescence band can be assigned to the ^3^T_2_(^3^F) → ^3^A_2_(^3^F) transition of octahedral Ni^2+^ ions, and 1550 nm emission is a result of the Er^3+^: ^4^I_13/2_ → ^4^I_15/2_ radiative transition. Normalized luminescence spectra enabled analysis of the influence of the heat treatment time on the I_1550nm_/I_1300nm_ intensity ratio. It is seen that the luminescence intensity of Ni^2+^ ions increases with the longer heat treatment time. The curve of I_1550nm_/I_1300nm_ vs. heat treatment time shows the smallest changes (0–2 h) in GGK glass with 20 ZnO (Figure 11). It might be the result of the faster nucleation of the ZnGa_2_O_4_ nanocrystals with incorporated Ni^2+^ ions. However, after 5 h, the I_1550nm_/I_1300nm_ ratio has stabilized and was estimated to be 14.5 (10ZnO), 15.6 (15ZnO), 17.8 (20ZnO). Moreover, a decrease in FWHM (full width at half maximum) of 1550 nm luminescence band from 77 nm (as-melted glasses) to 44 nm (HT for 5 h glass-ceramics) was observed in all investigated 0.1NiO/0.1Er_2_O_3_ -co-doped glasses. It suggests modifications in the site symmetry around erbium through the incorporation part of the Er^3+^ ions into the crystalline phase [44]. If only Ni^2+^ ions (not Er^3+^) are incorporated into ZnGa_2_O_4_ nanocrystals the distance between Ni^2+^ and Er^3+^ is elongated and energy transfer Ni^2+^ and Er^3+^ may be suppressed. This effect was observed in silicate glass ceramics [15]. Our research indicates that Ni^2+^ to Er^3+^ energy transfer occurs in the crystalline phase of germanate glass-ceramics.

Analysis of luminescence decay curves in glass-ceramics singly doped with Ni^2+^ and co-doped with Ni^2+^/Er^3+^ presented in Figure 12 enables to calculate the efficiency of Ni^2+^→Er^3+^ energy transfer according to the equation:

(2) $$\eta =1-\tau_{Ni-Er}^{}/\tau_{Ni}$$

where: $\tau_{Ni}^{}$ is the lifetime of Ni^2+^: ^3^T_2_(^3^F) in the presence of Er^3+^, $\tau_{Ni}$ is the lifetime of Ni^2+^: ^3^T_2_(^3^F) in singly Ni^2+^ -doped glass-ceramics. The lifetime of Ni^2+^: ^3^T_2_(^3^F) is characterized by double-exponential behavior. This effect suggests multiple side effects of Ni^2+^ and non-radiative multipolar interactions among Ni^2+^ [42]. The luminescence decay curves of the glass-ceramics singly doped with Ni^2+^ and co-doped with Ni^2+^/Er^3+^ were fitted by the sum of two exponential decay components from:

(3) $$I\left(t\right)=A_{1}\exp\left(-\frac{t}{\tau_{1}}\right)+{\;A}_{2}\exp\left(-\frac{t}{\tau_{2}}\right)$$

where τ_1_ and τ_2_ were short- and long-decay components, respectively. Parameters A_1_ and A_2_ were fitting constants. According to Equation (3), the average lifetime <τ> was given by:

(4) $$\langle \tau \rangle \;=\frac{A_{1}\tau_{1}^{2}+A_{2}\tau_{2}^{2}}{A_{1}\tau_{1}+A_{2}\tau_{2}}$$

According to Equation (4), the average lifetime of Ni^2+^:^3^T_2_(^3^F) in the fabricated GGK with 15ZnO glass-ceramics (HT@650 °C for 5 h) was calculated to be 433 μs (0.1NiO), and 349 μs (0.1NiO/0.1Er_2_O_3_). Thus, the efficiency of Ni^2+^ → Er^3+^ energy transfer (ET) was determined to be 19%. In the literature, in CaZrO_3_ nanocrystals co-doped with 0.2Ni/10Er (mol.%), the efficiency of the nickel to erbium energy transfer was estimated to be 86% [42]. In contrast, in silicate glass-ceramics with ZnGa_2_O_4_ nanocrystals (HT@ 850–950 °C) energy transfer between Ni^2+^ and Er^3+^ was suppressed [15]. It indicates that in the analyzed germanate glass-ceramics Ni^2+^ to Er^3+^ energy transfer occurs in the crystalline phase. Figure 13 presents the effect of the HT time GGK with 15 ZnO glass on the lifetime of Ni^2+^:^3^T_2_(^3^F) and Er^3+^: ^4^I_13/2_ metastable levels. Due to the incorporation of the nickel ions into ZnGa_2_O_4_ nanocrystals, the lifetime of the Ni^2+^ level increases with increasing the HT time and was calculated to be 62 μs (as-melted glass) and 433 μs (glass ceramics, HT–5 h). In silicate glass-ceramics with ZnGa_2_O_4_ and CaZrO_3_ nanocrystals, this value was 730 μs [12] and 600 μs [45], respectively. On the other hand, in silicate glass-ceramics with LiGa_5_O_8_ nanocrystals lifetime of Ni^2+:3^T_2_(^3^F) was 300 μs. In general, the Ni^2+^ lifetime has a positive correlation with the crystal field strength [12].

Analysis of the Er^3+^: ^4^I_13/2_ luminescence decay (Figure 13b) showed an increase in lifetime with increasing HT time (from 2.18 ms–as melted to 5.98 ms–HT 5h). It suggests that erbium ions are incorporated in the low-phonon energy ZnGaO_4_ nanocrystal phase. This effect is also consistent with the narrowing of the 1.55 μm luminescence band. Moreover, the Er^3+^: ^4^I_13/2_ lifetime increases with increasing the ZnO content (Figure 14), which could be the cause for more erbium ions in the nanocrystal phase. The Er^3+^: ^4^I_13/2_ lifetime was well fitted by a single-exponential curve which suggests one dominant structural position.

## 4. Conclusions

In summary, the effects of the ZnO content in transparent glass-ceramics containing ZnGa_2_O_4_ nanocrystals doped with Ni^2+^ and co-doped with Ni^2+^/Er^3+^ on structural and NIR luminescence properties were investigated. The controlled crystallization process enabled creation of ca. 11 nm size nanocrystals with incorporated Ni^2+^ions. According to the decomposed MIR spectra, it was found that the germanate-gallate networks of glass and glass-ceramics with 15 mol.% ZnO have existed as the [GeO_4_]/[GaO_4_], [GeO_6_]/[GaO_6_] structural units and connected through Ge, Ga–O–Ge, Ga bridges in [GeO_4_]/[GaO_4_] units. The structural changes of heat-treated precursor glasses showed local environment changes connected with the incorporation of parts of the Ni^2+^ and Er^3+^ ions into the crystalline phase of ZnGa_2_O_4_. This was in agreement with optical analysis. In particular, the broadband emission in the range of 1000–1650 nm (λ_exc_—808 nm) as a result of a superposition of the Ni^2+^: ^3^T_2_(^3^F) → ^3^A_2_(^3^F) octahedral Ni^2+^ ions and Er^3+^: ^4^I_13/2_ → ^4^I_15/2_ radiative transitions and energy transfer from Ni^2+^ to Er^3+^ with an efficiency of ca. 19%. Moreover, increasing the Er^3+^: ^4^I_13/2_ lifetime and narrowing the 1.55 μm emission band suggest that Er^3+^ ions are partially incorporated into ZnGa_2_O_4_ nanocrystals. It was also observed that ZnO content has a positive impact on the luminescent properties of GGK Ni^2+^/Er^3+^—co-doped glass-ceramics. The highest Ni^2+^ luminescence intensity and the longest lifetime of Er^3+^: ^4^I_13/2_ level have been obtained in GGK glass-ceramics with 20ZnO (mol.%). It is known that a longer lifetime of the metastable level is beneficial for obtaining high optical gain. It should be also emphasized that analyzed glass is thermally stable. Thus, investigated glass-ceramics are promising candidates for applications in broadband fiber emission sources of radiation or optical fiber amplifiers. Further research should be focused on the flattening of the emission in the whole 1000–1650 nm spectral range.

## Figures and Tables

**Table 1:** Parameters of component bands in MIR spectra.

Band	Position [cm^−1^]			Integral Intensity [%]		
	Sample					
	15ZnO_ as Melted	15ZnO_ 650 °C_3 h	15ZnO_ 650 °C_5 h	15ZnO_ as Melted	15ZnO_ 650 °C_3 h	15ZnO_ 650 °C_5 h
**A**	452	454	460	14	25	19
**B**	535	542	566	45	55	30
**C**	605	617	628	13	38	10
**D**	708	729	741	14	4	2
**E**	764	800	794	30	62	25
**F**	844	868	858	78	87	44
**G**	945	947	931	47	46	29
**H**	1036	1016	1011	44	34	29

**Table 2:** Assignment of component bands in MIR spectra [23,24,25,26,27,28,29,30,31,32,33,34].

Band	Assignment
**A**	symmetrical stretching bonds of Ge^[4]^, Ga^[4]^-O,-Ge^[4]^, Ga^[4]^ in germanano(gallo)oxygen four-membered rings
**B**	symmetrical stretching bonds of Ge^[4]^, Ga^[4]^-O-Ge^[4]^, Ga^[4]^ in the three-membered germanano(gallo)oxygen rings
**C**	symmetrical stretching bonds of Ge^[4]^, Ga^[4]^-O-Ge^[4]^, Ga^[4]^
**D**	symmetrical stretching bonds of Ge^[6]^, Ga^[6]^-O-Ge^[6]^, Ga^[6]^
**E**	asymmetrical stretching bonds of Ge^[4]^,Ga^[4]^-O-Ge^[4]^,Ga^[4]^ connecting [GeO]_4_/[GaO]_4_ tetrahedrons
**F**	asymmetrical stretching vibrations of the Ge-O-Ge, and Ga-O-Ga connecting [GeO_4_], [GaO_4_] tetrahedra
**G**	Ge–O–Ge or Ga–O–Ga stretching vibrations in [GeO_4_]/[GaO_4_]
**H**	stretching vibrations of the (Ge-O^−^) of GeO_4_ units

**Table 3:** Crystallography data for co-doped 15ZnO_650 °C_3h sample.

15ZnO_650 °C_3h						
No.	h	k	l	Position [°2Theta]	β [°2Theta]	d [nm]
1	1	1	1	18.40	1.1175	7.20
2	2	2	0	30.29	2.0217	4.07
3	3	1	1	35.65	2.4319	3.43
4	2	2	2	37.43	0.7497	11.19
5	4	0	0	43.52	0.7071	12.10
6	5	1	1	57.52	1.7866	5.07
7	4	4	0	63.32	0.8344	11.18
8	6	2	2	75.99	0.5634	17.89
9	4	4	4	80.12	0.6197	16.75

**Table 4:** Crystallography data for co-doped 15ZnO_650 °C_5h sample.

15ZnO_650 °C_5h						
No.	h	k	l	Position [°2Theta]	Β [°2Theta]	d [nm]
1	1	1	1	18.31	1.5736	5.11
2	2	2	0	30.25	1.9317	4.26
3	3	1	1	35.59	2.1037	3.97
4	2	2	2	37.38	0.6690	12.54
5	4	0	0	43.47	0.6311	13.55
6	5	1	1	57.47	2.3862	3.80
7	4	4	0	63.26	0.6667	14.00
8	6	2	2	75.98	0.5782	17.43
9	4	4	4	79.98	0.6889	15.05

## Data Availability

Not applicable.
